# SIRT1 in forebrain excitatory neurons produces sexually dimorphic effects on depression-related behaviors and modulates neuronal excitability and synaptic transmission in the medial prefrontal cortex

**DOI:** 10.1038/s41380-019-0352-1

**Published:** 2019-01-31

**Authors:** Yun Lei, Jiangong Wang, Dan Wang, Chen Li, Bin Liu, Xing Fang, Jingjing You, Ming Guo, Xin-Yun Lu

**Affiliations:** 1 Department of Neuroscience & Regenerative Medicine, Medical College of Georgia at Augusta University, Augusta, GA USA; 2 Department of Pharmacology, The University of Texas Health Science Center at San Antonio, San Antonio, TX USA; 3 Institute for Metabolic & Neuropsychiatric Disorders, Binzhou Medical University Hospital, Binzhou, Shandong China

**Keywords:** Neuroscience, Depression

## Abstract

Sirtuin 1 (SIRT1), an NAD^+^-dependent deacetylase, is a key regulator of cellular metabolism. Recent genome-wide association studies identified genetic variants of SIRT1 linked to major depressive disorders. SIRT1 is widely expressed in the brain; however, neuronal substrates that mediate SIRT1 action on depressive behaviors remain largely unknown. Here we show that selective deletion of SIRT1 in forebrain excitatory neurons causes depression-like phenotypes in male but not female mice. AAV-Cre-mediated SIRT1 knockdown in the medial prefrontal cortex (mPFC) of adult male mice induces depressive-like behaviors. Whole-cell patch-clamp recordings demonstrate that loss of SIRT1 decreases intrinsic excitability and spontaneous excitatory synaptic transmission in layer V pyramidal neurons in the prelimbic mPFC. Consistent with neuronal hypoexcitability, SIRT1 knockout reduces mitochondrial density and expression levels of genes involved in mitochondrial biogenesis and dynamics in the prelimbic mPFC. When a SIRT1 activator (SRT2104) is injected into the mPFC or lateral ventricle of wild-type mice, it reverses chronic unpredictable stress-induced anhedonia and behavioral despair, indicating an antidepressant-like effect. These results suggest that SIRT1 in mPFC excitatory neurons is required for normal neuronal excitability and synaptic transmission and regulates depression-related behaviors in a sex-specific manner.

## Introduction

Depression is a common mental disorder and a leading cause of disability. It is characterized by persistent symptoms that include depressed mood, anhedonia, suicidal thoughts, psychomotor retardation or agitation, changes in appetite and sleep patterns, loss of energy or increased fatigue, and poor concentration. Depressive disorders have a complex etiology, involving both genetic and environmental risk factors. Twin studies suggest that the heritability of major depressive disorders is 40–50% [1–4], while family studies indicate a 2–3-fold increased risk of major depression in the first-degree relatives [5]. However, owing to phenotypic and genetic heterogeneity, there has been limited success in identifying replicable genetic risk loci until recently with the first genome-wide significant results [6]. This large-scale genome-wide association study identified two genetic variants that contribute to the risk of major depressive disorder, one of which is sirtuin 1 (SIRT1) [6]. The association between the SIRT1 gene and depression has also been suggested by other genetic analysis [7, 8]. SIRT1 is an NAD(+)-dependent deacetylase that belongs to class III histone deacetylases and is highly conserved across species. It functions to deacetylate histones and affect gene expression epigenetically [9]. Additionally, it regulates acetylation of specific transcription factors and enzymes/proteins, thereby modulating their functions [10–13]. Owing to its ability to deacetylate a variety of substrates, SIRT1 is involved in a wide range of cellular processes, including energy metabolism, stress responses, mitochondrial biogenesis and turnover, cell proliferation, differentiation, and survival [13–15].

Several studies in animal models support an important role of SIRT1 in emotional regulation [16–20]. However, the exact neural network that mediates SIRT1 actions on depression remains unclear. SIRT1 is widely distributed in the brain with high levels in the hippocampus and cortex [15, 21–24], two brain regions rich in glutamatergic neurons [25]. Evidence suggests dysfunction of the glutamatergic system as a major pathological feature in mood disorders and a target for pharmacological intervention [26–28]. However, the functional role of SIRT1 in glutamatergic neurons and its impact on depression-related behaviors have not previously been investigated. In this study, we found that mice with conditional deletion of SIRT1 in forebrain glutamatergic neurons display a sex-specific depression-like phenotype, accompanied by reduced neuronal excitability and spontaneous excitatory synaptic transmission in pyramidal neurons in the medial prefrontal cortex (mPFC). While ablation of SIRT1 selectively in the mPFC of adult mice causes depressive behaviors, activation of SIRT1 in this region is sufficient to reverse depressive behaviors induced by chronic stress. Our findings suggest that SIRT1 in forebrain excitatory neurons functions as an important regulator of depression-related behaviors and a modulator of synaptic transmission and excitability of mPFC pyramidal neurons.

## Materials and methods

### Animals

Wild-type C57BL/6J, SIRT1^flox/flox^ (Stock No. 008041), and Emx1-ires-Cre knockin mice (Stock No. 005628) were purchased from Jackson Laboratory and maintained by breeding colonies. Animals were housed in groups of 3–5 under a 12/12-h light/dark cycle (lights on at 0700 h) with ad libitum access to food and water. Adult male and female mice were used for the experiments. All animal procedures were approved by the Institutional Animal Care and Use Committee of University of Texas Health Science Center at San Antonio and Binzhou Medical University Hospital.

### Generation of conditional SIRT1 knockout mice

SIRT1^flox/flox^ mice possess loxP sites flanking exon 4 of the SIRT1 gene, which encodes the conserved SIRT1 catalytic domain [29]. Emx1-ires-Cre knockin mice have an internal ribosome entry site (ires) and a Cre recombinase coding region inserted into the 3′ untranslated region of the mouse Emx1 gene. The Cre transgene is principally expressed in glutamatergic neurons in the hippocampus and neocortex including the mPFC [30–32]. SIRT1^flox/flox^ mice and Emx1-ires-Cre mice were maintained on a C57BL/6J background. To generate mice with SIRT1 knockout in forebrain excitatory neurons, SIRT1^flox/flox^ mice were crossed with Emx1-ires-Cre mice to produce SIRT1^flox/flox^, Emx1-ires-Cre (SIRT1^Emx1-KO^) mice. The PCR primers used for genotyping were as follows: Emx1-ires-Cre, forward-5′-CCAGCTAAACATGCTTCATCGTC-3′, reverse-5′-GGATTAACATTCTCCCACCGTCAG-3′; SIRT1, forward-5′-GGTTGACTTAGGTCTTGTCTG-3′, reverse-5′-CGTCCCTTGTAATGTTTCCC-3′.

### Drugs

SRT2104 (Selleck Chemicals LLC, Houston, TX, USA) was dissolved in dimethyl sulfoxide (DMSO) at a concentration of 5 mg/mL and diluted in phosphate-buffered saline (PBS) (8 g/L NaCl, 0.2 g/L KCl, 0.1 g/L MgCl_2_·6H_2_O, 0.133 g/L CaCl_2_·2H_2_O, 0.2 g/L KH_2_PO_4_, and 1.15 g/L Na_2_HPO_4_) to a final concentration of 0.05 µg/µL for intracranial infusion.

### RNA extraction and real-time PCR analysis

Adult male SIRT1^flox/flox^ and SIRT1^Emx1-KO^ mice were killed by decapitation and the mPFC, hippocampus, and hypothalamus were immediately dissected on ice. For the analysis of mRNA expression in the subregions of mPFC, brain tissue from adult male mice (12-week old) was sectioned in 80-μm-thick coronal sections in a cryostat (Leica Biosystems Inc., Buffalo Grove, IL). Prelimbic and infralimbic mPFC were dissected from the 80-µm-thick coronal brain sections (six sections from 1.98 mm to 1.54 mm from bregma) on ice according to the mouse brain atlas [33]. All samples were snap frozen in liquid nitrogen after dissection and stored at −80 °C until further processing for RNA extraction, as described below. Total RNA was extracted with the RNeasy Mini Kit (Qiagen, Germantown, MD). The cDNA was generated using the High-Capacity cDNA Reverse Transcription Kit (ThermoFisher, Waltham, MA) [34] and processed for real-time PCR quantification using the QuantStudio 3 real-time PCR system (ThermoFisher, Waltham, MA). The primer sequences used for amplification of SIRT1 and the genes involved in mitochondrial biogenesis and dynamics were as follows: SIRT1 exon 4, forward-5′-GATGCTGTGAAGTTACTGCAGGAGTG-3′, reverse-5′-GAGGGTCTGGGAGGTCTGGGAAG-3′ [35]; peroxisome proliferator-activated receptor gamma coactivator 1-alpha (PGC-1α), forward-5′-TGATGTGAATGACTTGGATACAGACA-3′, reverse-5′-GCTCATTGTTGTACTGGTTGGATATG-3′; mitofusin 1 (Mfn1), forward-5′-GCAGACAGCACATGGAGAGA-3′, reverse-5′-GATCCGATTCCGAGCTTCCG-3′; mitofusin 2 (Mfn2), forward-5′-TGCACCGCCATATAGAGGAAG-3′, reverse-5′-TCTGCAGTGAACTGGCAATG-3′; mitochondrial fission 1 protein (Fis1), forward-5′-CAAAGAGGAACAGCGGGACT-3′, reverse-5′-ACAGCCCTCGCACATACTTT-3′; dynamin-related protein 1 (Drp1), forward-5′-ATGCCAGCAAGTCCACAGAA-3′, reverse-5′-TGTTCTCGGGCAGACAGTTT-3′ [36]; β-tubulin, forward-5′-AGCAACATGAATGACCTGGTG-3′, reverse-5′-GCTTTCCCTAACCTGCTTGG-3′.

### Behavioral procedures

Mice were weighed weekly from 3 to 9 weeks of age. Food consumption (standard chow diet) was measured for 2 consecutive days. Average daily food intake was calculated and adjusted for body weight. Adult male and female mice (8–15 weeks) were used for behavioral tests. All behavioral procedures were performed in the late light cycle except the saccharin preference test and scored by experimenters who were blind to the genotypes or treatments. Mice were subjected to multiple behavioral tests spaced at least 3 days apart to decrease possible carryover effects from previous test. The order of behavioral tests was the same for each mouse; each mouse was tested once per test.

#### Saccharin/sucrose preference test

Mice were habituated to drinking water from two bottles for 1 week before testing. Mice were provided with a free choice of either drinking 0.01% saccharin solution or plain water. A computerized “lickometer” connected to an operant chamber (Med Associates Inc., Fairfax, VT) equipped with two bottles was used to determine the hedonic response of mice to sweet solutions. Mice were habituated to the operant chamber and water deprived overnight. Mice were offered a choice between saccharin solution and a lickometer recorded the number of licks to each sipper tube. The preference was calculated as the number of licks to the saccharin sipper tube divided by the total number of licks to all the sipper tubes times 100. The saccharin/sucrose preference was also measured in the home cage. Mice were provided with a free choice between a bottle containing saccharin (0.01%) or sucrose (1%) solution and a water bottle. Water and saccharin/sucrose intake was measured, and the preference for saccharin or sucrose was calculated by dividing the mass of saccharin solution consumed by the total mass of fluid intake.

#### Female urine sniffing test

This is a non-operant test to assess sex-related reward-seeking behavior based upon interest of male rodents in pheromonal odors from estrus female urine [37]. Male mice were subjected to the following test procedure: (1) 3-min exposure to the cotton tip dipped in water; (2) a 45-min interval; (3) 3-min exposure to the cotton tip dipped in fresh urine collected from female mice in the estrus phase; (4) a 45-min interval; and (5) 3-min exposure to the cotton tip dipped in fresh urine from male mice. The duration of female urine sniffing time was scored.

#### Forced swim test

Mice were placed in a clear Plexiglas cylinder (25 cm high; 10 cm in diameter) filled with 24 °C water to a depth of 15 cm. A charge-coupled device (CCD) camera positioned directly above the cylinder was used to record the behavior of each mouse for 6 min. The duration of immobility in the last 4 min was measured. Immobility was defined as no movement of the limb or body except those caused by respiration [34, 38, 39].

#### Locomotor activity

Mice were placed in the SuperFlex Fusion open field cages (40 × 40 × 30 cm^3^, Omnitech Electronics Inc., Columbus, OH) and allowed to freely explore for 30 min under the illuminated conditions. The movements of mice were monitored by infrared photosensors equipped on the cage and the total distance traveled was analyzed through the Fusion software (Omnitech Electronics Inc., Columbus, OH).

#### Learned helplessness test

The learned helplessness test was performed in a shuttle cage divided equally into two chambers with an auto-controlled guillotine door between the two chambers (Coulbourn Instruments, Holliston, MA) as previously described [38, 40]. Mice were subjected to 200 scrambled, inescapable foot shocks (0.3 mA shock amplitude, 2-s duration, 16-s average interval) over a 1 h session for 2 consecutive days to induce learned helplessness. Escape performance was tested 24 h after the last session in the same shuttle cage. Each mouse was given 30 shuttle escape trials with 25-s maximum duration and 30-s intervals. A sound cue and the shock took place at the same time as the guillotine door opened in the first five trials. For the remaining trials, the guillotine door opened 2 s after the shock was delivered. Each trial was terminated when the mouse crossed into the non-shock compartment. The latency to escape in each trial during the test were recorded automatically by the Graphic State software (Coulbourn Instruments Inc., Holliston, MA).

#### Chronic unpredictable stress (CUS)

Mice (9-week old) were subjected to a variety of stressors at different times of the day for 10 days. The stressors included 2-h restraint, 15-min tail pinch, 24-h constant light, 24-h wet bedding, and 45° cage tilt; 10-min inescapable foot shocks; and 30 min elevated platform and social isolation. Stress exposure was conducted in a procedure room, and the mice exposed to the CUS procedure were singly housed. Control mice were group housed and briefly handled daily in the housing room.

### Whole-cell patch-clamp recordings

Electrophysiological recordings were performed as previously described [41]. Male mice (8-week old) were anesthetized with isoflurane and brains were quickly transferred to an ice-cold solution (254 mM sucrose, 3 mM KCl, 2 mM MgCl_2_, 2 mM CaCl_2_, 1.25 mM NaH_2_PO_4_, 10 mM d-glucose, and 24 mM NaHCO_3_). Coronal brain slices (300 μm) containing the mPFC were prepared with a Leica VT1000S vibratome (Leica Microsystems) and allowed to recover at 30 °C for at least 1 h in an oxygenated (95% O_2_/5% CO_2_) artificial cerebrospinal fluid solution. Patch electrodes with tip resistances between 4 and 7 MΩ were filled with a potassium gluconate-based internal solution (120 mM potassium gluconate, 20 mM KCl, 2 mM MgCl_2_, 10 mM HEPES, 2 mM ATP, 0.25 mM GTP, and 0.1 mM EGTA adjusted to 7.4 and osmolarity of 295 mOsm). Pyramidal neurons in the layer V of mPFC were visualized with a 40× water-immersion lens and recorded with the Multiclamp 700 A amplifier (Molecular Devices, Sunnyvale, CA). To investigate the firing properties of these neurons, current-clamp recordings were made from a −70 mV holding current and incremental stepwise positive current injections (15 pA) for a 0.5 s duration. Spontaneous excitatory postsynaptic currents (sEPSCs) were recorded in the voltage-clamp mode with membrane potentials held at −70 mV in the presence of picrotoxin (100 μM). Tetrodotoxin (TTX, 1 μM) was added to block action potential (AP) formation and its propagation for the recording of miniature EPSCs. For neuronal excitability in response to current injections, pyramidal neurons in the prelimbic and infralimbic mPFC were recorded on brain slices from the same mice. For sEPSCs and miniature EPSCs, pyramidal neurons in the prelimbic and infralimbic mPFC were recorded from different mice.

### Electron microscopic detection of mitochondria

Male mice (8-week old) were transcardially perfused with 0.1 M PBS followed by 2% paraformaldehyde (PFA) and 2% glutaraldehyde. Brains were removed and coronal brain sections were sliced at 1 mm with Leica VT1000S vibratome (Leica Microsystems, Wetzlar, Germany). Then the brain slices were modified into 1 mm^3^ tissue blocks containing the mPFC. The following electron microscopic procedure was performed by the Pathology Electron Microscopy Facility of the University of Texas Health Science Center at San Antonio using the standard techniques as described by others [42, 43]. Briefly, the brain tissue blocks were fixed in a phosphate-buffered solution containing 4% formaldehyde and 1% glutaraldehyde for 2 h and further fixed in 1% Zetterqvist’s buffered osmium tetroxide for 90 min at room temperature. After washing, brain tissues were dehydrated with a series of ethanol (70%, 95%, 100%, 100%), cleared twice in propylene oxide, and infiltrated with propylene oxide–resin (v/v = 1:1) followed by 100% resin buffer for 90 min. Tissues were then embedded in plastic resin. Orientation and section quality was checked with 1-μm-thick sections stained with 1% toluidine blue under a light microscope. Ultrathin sections (90 nm) were cut and collected on standard copper grids counterstained with 1% uranyl acetate for 10 min with Reynold’s lead citrate and viewed in a FEI Tecnai G2 Spirit transmission electron microscope. The images were achieved with AMT’s CCD imaging system. The sections containing a visible nucleus were randomly selected and analyzed by an experimenter blind to the genotype of mice. The total number of mitochondria contained within the cytoplasmic surface area was quantified using ImageJ. The total area of cytoplasm was measured. Mitochondrial density was estimated by dividing the total number of mitochondria by the cytoplasmic area and was expressed as the number of mitochondria per square micrometers of cytoplasm.

Separate cohorts of SIRT1^flox/flox^ and SIRT1^Emx1-KO^ mice were used for behavioral phenotyping, whole-cell patch-clamp recordings, electron microscopic detection of mitochondria, and real-time PCR analysis of expression of the genes involved in mitochondrial biogenesis and dynamics.

### Stereotaxic surgery, microinjection, and cannulation

**Fig. 1:**
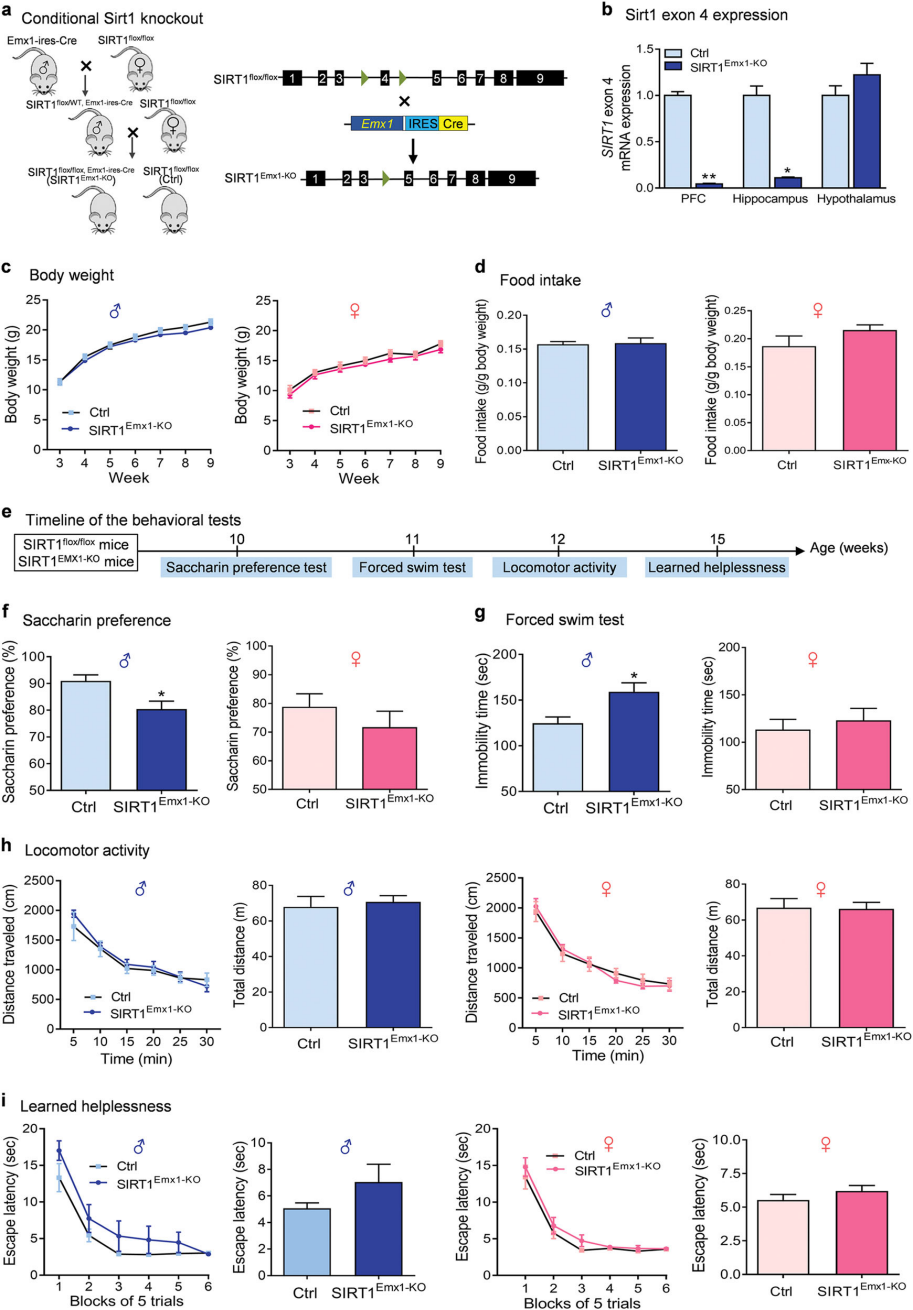

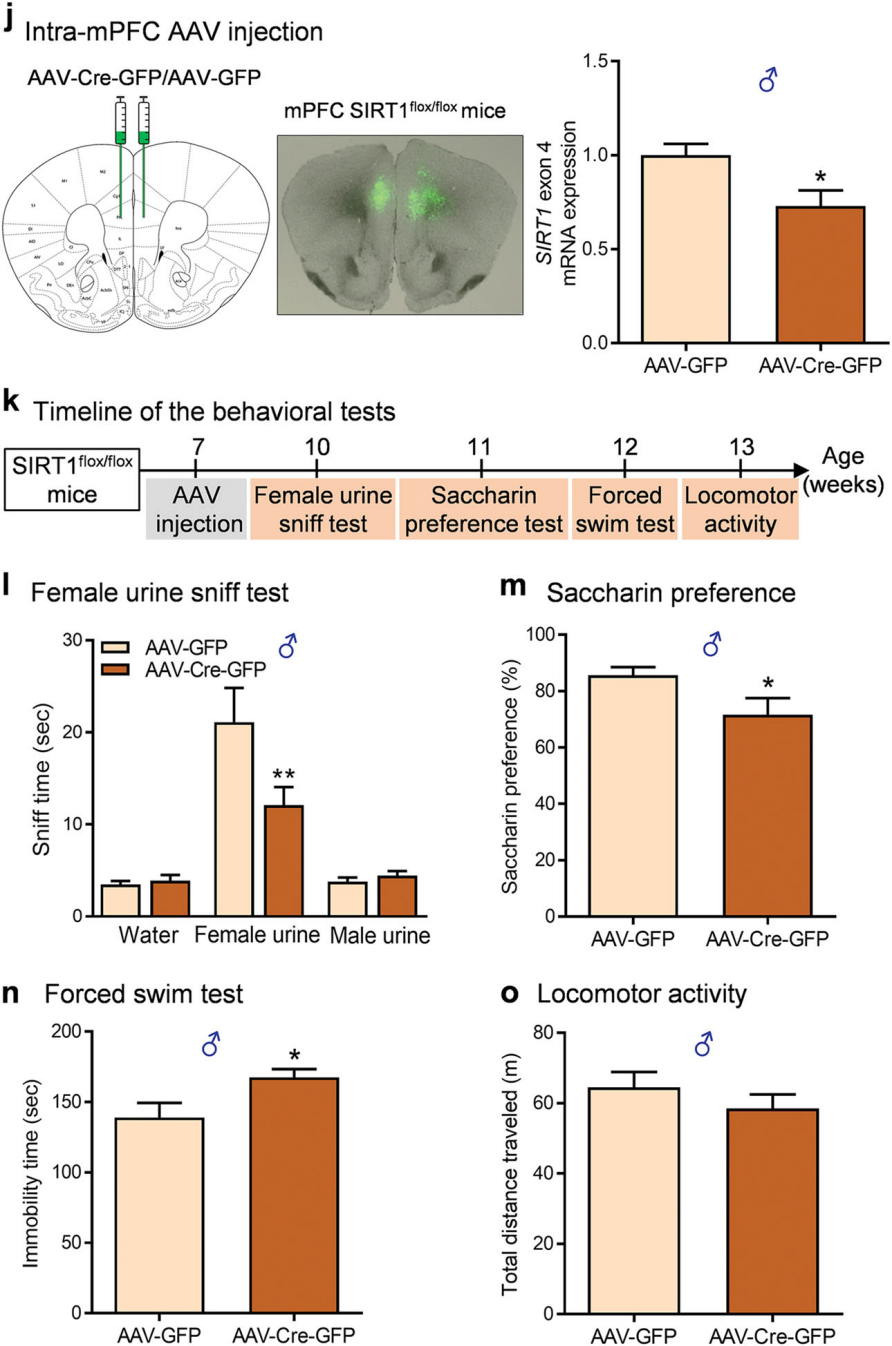
Ablation of sirtuin 1 (SIRT1) in forebrain excitatory neurons induces depressive-like behaviors. **a** Left, a schematic of the breeding strategy used to generate mice lacking SIRT1 activity in forebrain excitatory neurons (SIRT1^Emx1-KO^); right, schematic diagram of generating conditional SIRT1 knockout mice from breeding Emx1-ires-Cre mice with mice carrying homozygous SIRT1 exon 4-floxed allele. **b** Verification of deletion of SIRT1 exon 4 in SIRT1^Emx1-KO^ mice. Real-time quantitative PCR showing reduced mRNA expression levels of exon 4 in the prefrontal cortex (PFC) and hippocampus but not in the hypothalamus of SIRT1^Emx1-KO^ mice compared to control SIRT1^flox/flox^ (Ctrl) mice. *n* = 5 per group. **c**–**i** Phenotypic characterization of SIRT1^Emx1-KO^ mice. **c** Body weight. Male mice: Ctrl, *n* *=* 15; SIRT1^Emx1-KO^, *n* = 20; female mice: Ctrl, *n* = 8; SIRT1^Emx1-KO^, *n* = 6. **d** Food intake. Male mice: Ctrl, *n* = 6; SIRT1^Emx1-KO^, *n* = 5; female mice: Ctrl, *n* = 7; SIRT1^Emx1-KO^, *n* = 11. **e** Timeline of behavioral tests. **f** Saccharin preference test. Male mice: Ctrl, *n* = 9; SIRT1^Emx1-KO^, *n* = 9; female mice: Ctrl, *n* = 11; SIRT1^Emx1-KO^, *n* = 10. **g** Forced swim test. Male mice: Ctrl, *n* = 9; SIRT1^Emx1-KO^, *n* = 10; female mice: *n* = 11 per group. **h** Locomotor activity. Left, middle-right: time course of locomotor activity over the 30-min test session; middle-left and right: total distance traveled in 30 min. Male mice: Ctrl, *n* = 9; SIRT1^Emx1-KO^, *n* = 10; female mice: Ctrl, *n* = 11; SIRT1^Emx1-KO^, *n* = 10. **i** Learned helplessness test. Left, middle-right: Mean escape latency over blocks of five trials; middle-left and right: the average of escape latency in all trials. Male mice: Ctrl, *n* = 10; SIRT1^Emx1-KO^, *n* = 10; female mice: Ctrl, *n* = 11; SIRT1^Emx1-KO^, *n* = 10. **j**–**o** Ablation of SIRT1 in the medial PFC (mPFC) induced by AAV-Cre-GFP in adult male SIRT1^flox/flox^ mice. **j** Left, schematic illustration of stereotaxic injection of AAV-Cre-GFP or AAV-GFP vectors in the mPFC; middle, representative image showing GFP expression at the injection sites; right, AAV-Cre-mediated knockdown of SIRT1 in the mPFC. *n* = 4 male mice per group. **k** Timeline of behavioral tests. **l** Female urine sniffing test. AAV-GFP, *n* = 14; AAV-Cre-GFP, *n* = 16. **m** Saccharin preference test. AAV-GFP, *n* = 12; AAV-Cre-GFP, *n* = 12. **n** Forced swim test. AAV-GFP, *n* = 9; AAV-Cre-GFP, *n* = 10. **o** Locomotor activity. Total distance traveled in 30 min. AAV-GFP, *n* = 14; AAV-Cre-GFP, *n* = 16. *P < 0.05, **P < 0.01 compared with the Ctrl or AAV-GFP group

Stereotaxic surgery was performed under anesthesia as previously described [38, 41, 44]. For intra-mPFC viral injection, AAV5-CMV-Cre-GFP (in the following referred to as AAV-Cre-GFP) containing the genes for Cre recombinase and green fluorescent protein (GFP) and control AAV5-CMV-GFP (in the following referred to as AAV-GFP) containing the gene for GFP alone with titers >1 × 10^12^ vg/mL (UNC Vector Core, Chapel Hill, NC) were injected bilaterally into the mPFC (coordinates: anterior–posterior (AP) = 1.8 mm, medial–lateral (ML) = ± 0.4 mm, dorsal–ventral (DV) = −2.6 mm from the bregma) of adult male SIRT1^flox/flox^ mice (7-week old). A total volume of 0.5 μL adeno-associated viral (AAV) vectors (per side) was delivered at a rate of 0.10 μL/min with a 33-gauge stainless steel injector connected to a UMP3 micro syringe pump (World Precision Instruments, Sarasota, FL). Behavioral tests were performed 3 weeks after AAV injection. The injection sites were verified in each animal at the end of the experiments (Fig. 1j). Mice with “missed” injections were excluded from statistical analysis.

For intra-mPFC microinjection of the SIRT1 activator SRT2104, a 26-gauge double-guide cannula (Plastics One) was implanted 1 mm above the mPFC (coordinates: AP = 1.8 mm, ML = ± 0.4 mm, DV = −1.6 mm from the bregma) of adult male C57BL/6J mice (8-week old). Following surgery, mice were individually housed and allowed to recover for 7 days before the chronic stress procedure. Microinjections were performed on freely moving mice in their home cage. On the experimental day, a bilateral injection cannula (33-gauge) connected to a 5-μL syringe was inserted into the guide cannula and extended 1 mm beyond the tip. SRT2104 (0.05 µg/µL) or vehicle (1% DMSO in PBS) were infused into the mPFC in a volume of 0.2 µL over 2 min using an infusion pump (KD Scientific Inc., Holliston, MA). Injectors were held in place for an additional 5 min after the infusion to avoid backflow. Mice were given three infusions of SRT2104 within 24 h (23, 3, and 1 h) before behavioral testing. This sub-chronic drug treatment paradigm was based upon three injections over a 24-h period for testing antidepressants that was first reported by Porsolt et al. [45]. The purpose of multiple SRT2104 injections was to induce sufficient activation of SIRT1.

For intracerebroventricular (ICV) injection of the SIRT1 activator SRT2104, a guide cannula (C315GS, Plastics One) was implanted into the lateral ventricle (coordinates: AP = −0.2 mm, ML = 1.1 mm, DV = −1.7 mm from the bregma) of adult male C57BL/6J mice (8-week old) as described previously [38]. The injection cannula (33-gauge) connected to a 5-μL syringe was inserted into the guide cannula and extended 1 mm beyond the tip. SRT2104 (0.03 µg/µL) or vehicle (0.67% DMSO in PBS) were infused into the lateral ventricle in a volume of 1.0 µL over 2 min using an infusion pump. Injectors were held in place for an additional 5 min after the infusion. Mice were given two infusions of SRT2104 within 24 h (23 and 3 h) before behavioral testing.

### Immunohistochemistry

To detect protein expression of SIRT1 in the brain, immunohistochemistry was performed as described previously [46–48]. Briefly, adult male C57BL/6J mice (8-week old) were transcardially perfused under anesthesia through the ascending aorta using 0.1 M PBS followed by 4% PFA in PBS. The brains were removed and fixed overnight in 4% PFA and then transferred to 30% sucrose in PBS. Brains were cut into 40-μm coronal sections on a cryostat and stored in cryoprotectant (30% sucrose, 30% ethylene glycol, 1% polyvinyl pyrrolidone, 0.05 M sodium phosphate buffer) until processing for immunohistochemistry. Free floating sections were first treated with 1% hydrogen peroxide in PBS to quench the endogenous peroxidase. The tissue was then incubated in immuno blocking buffer (3% goat serum, 1% bovine serum albumin, 0.3% triton-X 100 in PBS) for 1 h at room temperature followed by rabbit anti-SIRT1 (1:1000, #07–131, EMD Millipore, Burlington, MA, USA) in blocking solution for 48 h at 4 °C. After rinsing in PBS buffer, the sections were incubated with goat anti-rabbit IgG secondary antibody conjugated to horseradish peroxidase (1:500) at room temperature for 4 h. The sections were washed in PBS and developed with the DAB Peroxidase Substrate Kit (Vector Laboratories, Burlingame, CA). Olympus BX51 microscope (Olympus Scientific Solutions America, Waltham, MA, USA) was used to visualize the immunostaining in the mPFC and capture images.

### Statistical analysis

All results are presented as mean ± s.e.m. (standard error of mean). Shapiro–Wilk test and *F* test were used to test the normality and equal variance assumptions, respectively. For normally distributed data, two-tailed *t* tests were used to assess differences between two experimental groups with equal variance. For a two-sample comparison of means with unequal variances, two-tailed *t* tests with Welch’s correction were used. One-way analyses of variance (ANOVAs) followed by Sidak post hoc tests were used for analysis of three or more groups. For non-normally distributed data, Mann–Whitney *U* tests were performed to compare two groups. For analysis of three or more groups with non-normally distribution, the Kruskal-Wallis test followed by Dunn's multiple comparisons test was used. For locomotor activity, the escape latency in the learned helplessness test, and the number of APs elicited by current injections, two-way repeated-measures ANOVAs followed by Bonferroni tests were used. *P* < 0.05 was considered statistically significant.

## Results

### Generation of mice lacking SIRT1 in forebrain excitatory neurons

To determine the functional role of SIRT1 in forebrain excitatory neurons, we generated mice lacking SIRT1 activity in forebrain glutamatergic neurons by crossing SIRT1^flox/flox^ mice, in which exon 4 of the SIRT1 gene is flanked by loxP sites, with Emx1-ires-Cre mice having the endogenous *Emx1* locus directing expression of Cre recombinase to the vast majority of glutamatergic neurons in the neocortex and hippocampus (Fig. 1a) [31, 49]. Although both glia and glutamatergic neurons are derived from the Emx1 lineage, Cre activity in this line of Emx1-ires-Cre knockin mice appeared to be very weak in glial cells [31]. Moreover, within the adult brain, SIRT1 was found to be prominent in neurons [21, 23, 24]. Thus Emx1-Cre-mediated deletion of SIRT1 most likely occur in excitatory neurons rather than in glia. Mice with both floxed alleles and Cre transgene, i.e., SIRT1^flox/flox^, Emx1-ires-Cre (hereafter referred as SIRT1^Emx1-KO^ mice), and SIRT1^flox/flox^ as control were used for the experiments. Ablation of SIRT1 exon 4 was confirmed by real-time quantitative PCR in the PFC and hippocampus, whereas the expression levels of exon 4 in the hypothalamus remained unchanged (Fig. 1b, Kruskal–Wallis test, *P* < 0.001).

Studies have shown that brain SIRT1 regulates body weight and food intake [50, 51]. Evidence suggests that hippocampal and mPFC neural processes impact energy balance and food-motivated behavior [52]. We therefore examined whether body weight and feeding were altered in mice lacking SIRT1 activity in hippocampal and cortical glutamatergic neurons. Both male and female SIRT1^Emx1-KO^ mice exhibited normal growth of their body weight (Fig. 1c; genotype: *F*_(1,33)_ = 1.174, *P* = 0.287; time: *F*_(6,198)_ = 473.4, *P* < 0.001; genotype × time: *F*_(6,198)_ = 1.445, *P* = 0.199 for male mice; genotype: *F*_(1,12)_ = 0.900, *P* = 0.361; time: *F*_(6,72)_ = 106.6, *P* < 0.001; genotype × time: *F*_(6,72)_ = 0.321, *P* = 0.924 for female mice). As to feeding behavior, the intake of standard chow was measured on 2 consecutive days in adult mice (8–12 weeks). There was no significant difference between SIRT1^Emx1-KO^ and control mice (Fig. 1d; *t*_(9)_ = 0.205, *P* = 0.842 for male mice; *t*_(16)_ = 1.546, *P* = 0.142 for female mice).

### Male, but not female, SIRT1^***Emx1***-***KO***^ mice exhibit a depression-like phenotype

To determine whether inactivation of SIRT1 in forebrain excitatory neurons affects depression-related behaviors, SIRT1^Emx1-KO^ mice and their littermate controls were assessed using different behavioral tests. Anhedonia is a core symptom of depression, which can be evaluated in rodents using the sucrose preference test [53–55]. Since SIRT1 functions as a metabolic sensor, the caloric value of sucrose may confound the sucrose preference test results. Thus we used non-caloric sweetener saccharin to measure hedonic response. Saccharin preference was assessed with a computerized “lickometer.” We found that male SIRT1^Emx1-KO^ mice exhibited a significant decrease in preference for a 0.01% saccharin solution, when compared to wild-type littermate controls (Fig. 1f; *t*_(16)_ = 2.786, *P* = 0.013). However, female SIRT1^Emx1-KO^ mice displayed normal preference for saccharin (Fig. 1f; *t*_(19)_ = 0.957, *P* = 0.351). Additionally, male SIRT1^Emx1-KO^ mice showed significantly increased immobility time in the forced swim test (Fig. 1g; *t*_(17)_ = 2.847, *P* = 0.011), whereas female mutant mice displayed normal immobility in this test (Fig. 1g; *t*_(20)_ = 0.557, *P* = 0.584). To examine whether increased immobility may result from a decrease in non-specific motor activity, locomotor activity was measured in an open field chamber and showed no genotype difference in both male mice (Fig. 1h; distance travelled: genotype, *F*_(1,17)_ = 0.178, *P* = 0.678; time, *F*_(5,85)_ = 48.22, *P* < 0.001; genotype × time, *F*_(5,85)_ = 0.848, *P* = 0.520; total distance: *t*_(17)_ = 0.422, *P* = 0.678) and female mice (Fig. 1h; distance travelled: genotype, *F*_(1,19)_ = 0.008, *P* = 0.928; time, *F*_(5,95)_ = 80.37, *P* < 0.001; genotype × time, *F*_(5,95)_ = 0.685, *P* = 0.636; total distance: *t*_(19)_ = 0.092, *P* = 0.928). These results indicate that loss of SIRT1 in cortical and hippocampal excitatory neurons causes sexually dimorphic anhedonia and despair behavioral phenotypes. Moreover, we tested the mice in a learned helplessness paradigm. After exposure to inescapable shock stress, mice were assessed for a coping deficit in avoidance–escape performance. The escape latency in SIRT1^Emx1-KO^ was comparable to that in littermate control mice (Fig. 1i; male mice: genotype, *F*_(1,18)_ = 2.004, *P* = 0.174; block, *F*_(5,90)_ = 54.03, *P* < 0.001; genotype × block, *F*_(5,90)_ = 1.02, *P* = 0.411; average escape latency, Mann–Whitney test, *P* = 0.089; female mice: genotype, *F*_(1,19)_ = 1.551, *P* = 0.228; block, *F*_(5,95)_ = 65.73, *P* < 0.001; genotype × block, *F*_(5,95)_ = 0.323, *P* = 0.898; average escape latency, Mann–Whitney test, *P* = 0.072).

### AAV-Cre-mediated SIRT1 knockdown in the mPFC of adult male mice causes depression-like behaviors

The mPFC plays a critical role in the pathophysiology of depression [56]. We hypothesized that the mPFC mediates SIRT1 action on depression-like behaviors. We used Cre recombinase-expressing AAV (AAV-Cre-GFP) vectors to selectively ablate the loxP-flanked SIRT1 gene. The region-specific SIRT1 knockdown in the mPFC was achieved by bilateral intra-mPFC injection of AAV-Cre-GFP in adult male SIRT1^flox/flox^ mice. Three weeks later, we confirmed AAV-mediated knockdown of the SIRT1 exon 4 in the mPFC (Fig. 1j, *t*_(6)_ = 2.619, *P* = 0.040). The relatively low knockdown (~27%) is largely due to the restricted microinjection site and the mPFC dissection including tissue/cells that were not targeted by AAV vectors. The behavioral consequences of ablation of SIRT1 in the mPFC were assessed using the following tests. We used the female urine sniffing test to assess sex-related reward seeking behavior, which is based upon interest of male rodents in pheromonal odors from estrus female urine [37]. We found that AAV-Cre-GFP-injected mice spent less time sniffing female urine compared to AAV-GFP-injected control mice, whereas they spent similar amounts of time sniffing water or male urine (Fig. 1l; vector, *F*_(1,84)_ = 3.622, *P* = 0.060; sniffing object, *F*_(2,84)_ = 36.73, *P* < 0.001; vector × sniffing object, *F*_(2,84)_ = 5.214, *P* = 0.007). In the saccharin preference test, mice injected with AAV-Cre-GFP showed a decrease in preference for 0.01% saccharin solution compared with AAV-GFP-injected control mice (Fig. 1m, *t* test with Welch’s correction, *P* = 0.050). These results indicate that ablation of SIRT1 led to an anhedonic phenotype with the deficits in both consummatory and motivational aspects of reward-related behaviors. In the forced swim test, AAV-Cre-GFP injection in the mPFC increased immobility time (Fig. 1n, *t*_(17)_ = 2.499, *P* = 0.023) without affecting locomotor activity (Fig. 1o, *t*_(28)_ = 1.026, *P* = 0.314), suggesting a behavioral despair phenotype.

### Ablation of SIRT1 decreases neuronal excitability and excitatory synaptic transmission in layer V pyramidal neurons in the prelimbic mPFC

**Fig. 2:**
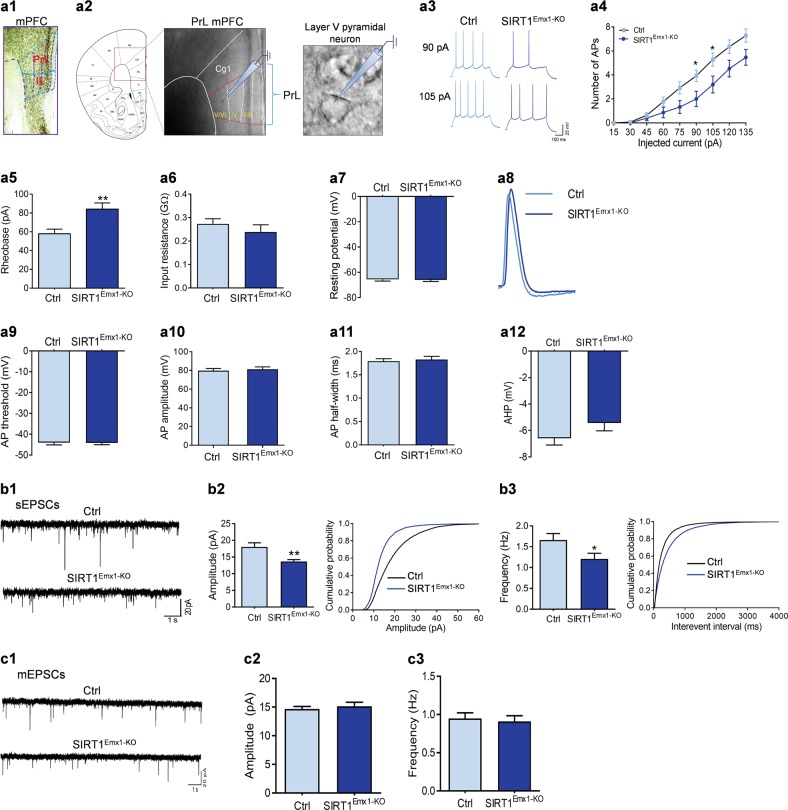
Sirtuin 1 (SIRT1) is required for normal neuronal excitability and excitatory synaptic transmission in layer V pyramidal neurons in the prelimbic medial prefrontal cortex (mPFC). **a1** SIRT1 immunoreactivity in the mPFC. PrL prelimbic, IL infralimbic. **a2** Schematic illustration of recordings from layer V pyramidal neurons in the prelimbic mPFC in acute brain slices. **a3**–**a12** Intrinsic neuronal excitability. **a3** Depolarizing current injections evoke trains of action potentials (APs) in PrL PFC pyramidal neurons. **a4** Number of APs elicited by depolarizing current injection. **a5** Rheobase current. **a6** Input resistance. **a7** Resting membrane potential. **a8** The first AP evoked by current injection. **a9** AP threshold. **a10** AP amplitude. **a11** AP half-width. **a12** Afterhyperpolarization (AHP). *n* = 14 neurons from 4 male control SIRT1^flox/flox^ (Ctrl) mice; *n* = 15 neurons from 5 male SIRT1^Emx1-KO^ mice. **b1**–**b3** Spontaneous excitatory postsynaptic currents (sEPSCs). **b1** Representative sEPSC traces. **b2** Left, average sEPSC amplitude of recorded pyramidal neurons from two genotype groups; right, cumulative probability plots for sEPSC amplitudes. **b3** Left, average of sEPSC frequency; right, cumulative probability plots for the interevent interval for sEPSCs. *n* = 22 neurons from 4 male Ctrl mice; *n* = 23 neurons from 4 male SIRT1^Emx1-KO^ mice. **c1**–**c3** Miniature EPSCs (mEPSCs). **c1** Representative mEPSC traces. **c2** Average mEPSC amplitude. **c3** Average mEPSC frequency. *n* = 27 neurons from 5 male Ctrl mice; *n* = 23 neurons from 5 male SIRT1^Emx1-KO^ mice. *P < 0.05, **P < 0.01 compared with the Ctrl group

The glutamatergic outputs from the mPFC originate primarily from layer V pyramidal neurons [57]. Direct optogenetic stimulation of layer V pyramidal neurons produces a robust antidepressant-like response [58], suggesting a critical role of the activity of layer V pyramidal neurons in modulating depressive behavior. Thus, we determined whether the depressive phenotype in male SIRT1^Emx1-KO^ mice is associated with alterations in intrinsic neuronal excitability and excitatory synaptic transmission in mPFC layer V pyramidal neurons. First, we confirmed the expression of SIRT1 protein in the mPFC, including the prelimbic and infralimbic subregions using immunohistochemistry (Fig. 2a1). Given opposing influences of these two subregions on diverse emotional processes [59–61], we performed whole-cell patch-clamp recordings on layer V pyramidal neurons in the prelimbic and infralimbic mPFC, respectively (Figs. 2a2 and  3a1).

**Fig. 3:**
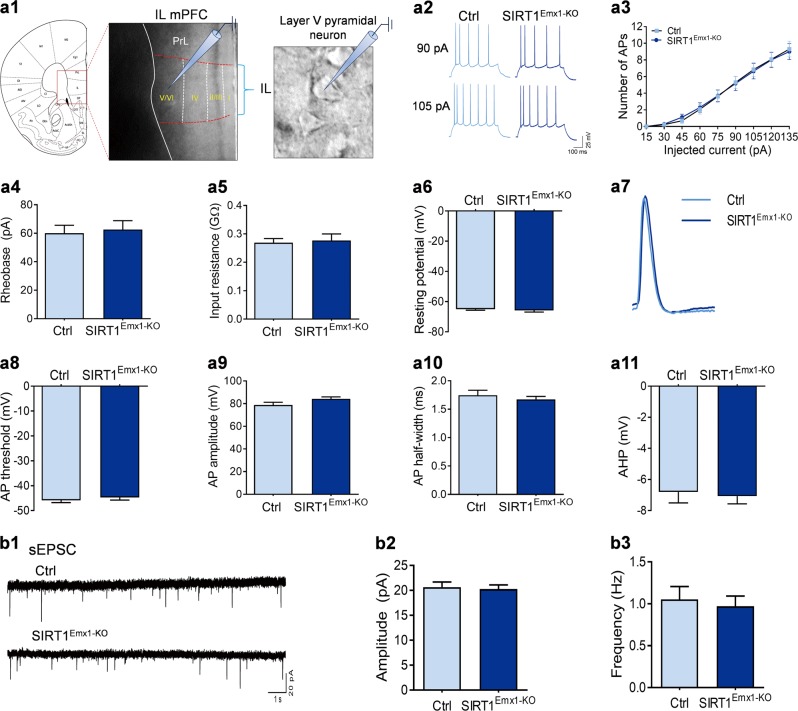
Loss of sirtuin 1 (SIRT1) activity has no effect on neuronal excitability and excitatory synaptic transmission in layer V pyramidal neurons in the infralimbic (IL) medial prefrontal cortex (mPFC). **a1** Schematic illustration of recordings from layer V pyramidal neurons in the infralimbic mPFC in acute brain slices. **a2**–**a11** Intrinsic neuronal excitability. **a2** Depolarizing current injections evoke trains of action potentials (APs) in IL PFC pyramidal neurons. **a3** Number of APs elicited by depolarizing current injection. **a4** Rheobase current. **a5** Input resistance. **a6** Resting membrane potential. **a7** The first AP evoked by current injection. **a8** AP threshold. **a9** AP amplitude. **a10** AP half-width. **a11** Afterhyperpolarization (AHP). *n* = 13 neurons from 4 male control SIRT1^flox/flox^ (Ctrl) mice mice; *n* = 17 neurons from 5 male SIRT1^Emx1-KO^ mice. **b1–b3** Spontaneous excitatory postsynaptic currents (sEPSCs). **b1** Representative sEPSC traces. **b2** Average sEPSC amplitude of recorded pyramidal neurons. **b3** Average sEPSC frequency. *n* = 19 neurons from 3 male Ctrl mice; *n* = 17 neurons from 3 male SIRT1^Emx1-KO^ mice

### Prelimbic mPFC layer V pyramidal neurons

To evaluate intrinsic excitability, the firing responses of layer V pyramidal neurons in the prelimbic mPFC to depolarizing current injections were analyzed under a current-clamp mode. Neurons from SIRT1^Emx1-KO^ mice generated fewer APs than neurons from wild-type littermate control mice in response to the same amount of current injections (Fig. 2a3, a4; current: *F*_(8,216)_ = 138.7, *P* < 0.001; genotype: *F*_(1,27)_ = 4.507, *P* = 0.043; current × genotype: *F*_(8,216)_ = 4.612, *P* < 0.001). The rheobase, the minimum injecting current to elicit AP, was significantly increased in prelimbic mPFC layer V pyramidal neurons from SIRT1^Emx1-KO^ mice (Fig. 2a5, *t*_(27)_ = 3.112, *P* = 0.004). Other membrane properties including input resistance (Fig. 2a6, Mann–Whitney test, *P* = 0.136) and resting membrane potential (Fig. 2a7, *t*_(27)_ = 0.205, *P* = 0.834) showed no difference between the two genotypes. In addition, we analyzed the properties of the first AP evoked by current injection (Fig. 2a8), including threshold (Fig. 2a9, *t*_(28)_ = 0.127, *P* = 0.900), amplitude (Fig. 2a10, *t*_(28)_ = 0.475, *P* = 0.639), half-width (Fig. 2a11, Mann–Whitney test, *P* = 0.943), and afterhyperpolarization (AHP; Fig. 2a12, *t*_(28)_ = 1.453, *P* = 0.157), which did not differ between genotypes.

To determine the impact of loss of SIRT1 on excitatory synaptic transmission in layer V pyramidal neurons, we recorded spontaneous excitatory postsynaptic responses (Fig. 2b1). The amplitude of sEPSCs of prelimbic mPFC layer V pyramidal neurons from SIRT1^Emx1-KO^ mice was significantly decreased when compared to the neurons from control mice, as shown by the reduced average amplitude (unpaired *t* test with Welch’s correction, *P* = 0.003) and the left-shifted cumulative frequency distribution of peak amplitude of all events (Kolmogorov–Smirnov test, *P* < 0.001) (Fig. 2b2). The average frequency of recorded neurons was also significantly decreased (Mann–Whitney test, *P* = 0.024), and the cumulative frequency distribution of inter-event intervals was right-shifted in SIRT1^Emx1-KO^ mice (Kolmogorov–Smirnov test, *P* < 0.001) (Fig. 2b3). To determine whether the changes in postsynaptic responses is dependent on APs, we recorded miniature EPSCs in the presence of 1 µM TTX to block AP formation and its propagation (Fig. 2c1). The average amplitude and frequency of recorded neurons did not reveal any significant difference between SIRT1^Emx1-KO^ and control mice (Fig. 2c2; amplitude, unpaired *t* test with Welch’s correction, *P* = 0.577; frequency, Mann–Whitney test, *P* = 0.710).

### Infralimbic mPFC layer V pyramidal neurons

Similar electrophysiological recordings were performed on layer V pyramidal neurons in the infralimbic mPFC. In contrast to prelimbic mPFC layer V pyramidal neurons, intrinsic membrane properties of infralimbic mPFC layer V pyramidal neurons were not affected by loss of SIRT1. The AP responses in these neurons evoked by depolarizing current injections were comparable between SIRT1^Emx1-KO^ and control mice (Fig. 3a2, a3; current: *F*_(8,224)_ = 130.8, *P* < 0.001; genotype: *F*_(1,28)_ = 0.0001, *P* = 0.991; current × genotype: *F*_(8,224)_ = 0.138, *P* = 0.998). No significant changes were observed in other membrane properties, including rheobase (Fig. 3a4, Mann–Whitney test, *P* = 0.999), input resistance (Fig. 3a5, unpaired *t* test with Welch’s correction, *P* = 0.773), and resting membrane potential (Fig. 3a6, Mann–Whitney test, *P* = 0.288). The analysis of the first AP revealed no difference between the two genotypes (Fig. 3a7–a11; AP threshold, Mann–Whitney test, *P* = 0.207; AP amplitude, *t*_(29)_ = 1.885, *P* = 0.069; AP half-width, Mann–Whitney test, *P* = 0.741; AHP, *t*_(29)_ = 0.320, *P* = 0.751). Next we recorded and analyzed sEPSCs in layer V pyramidal neurons in the infralimbic mPFC, which revealed no significant difference in the average amplitude (Fig. 3b2, *t*_(34)_ = 0.283, *P* = 0.779) and frequency (Fig. 3b3, Mann–Whitney test, *P* = 0.994) between the two genotypes.

### Ablation of SIRT1 impairs mitochondrial biogenesis in mPFC neurons

**Fig. 4:**
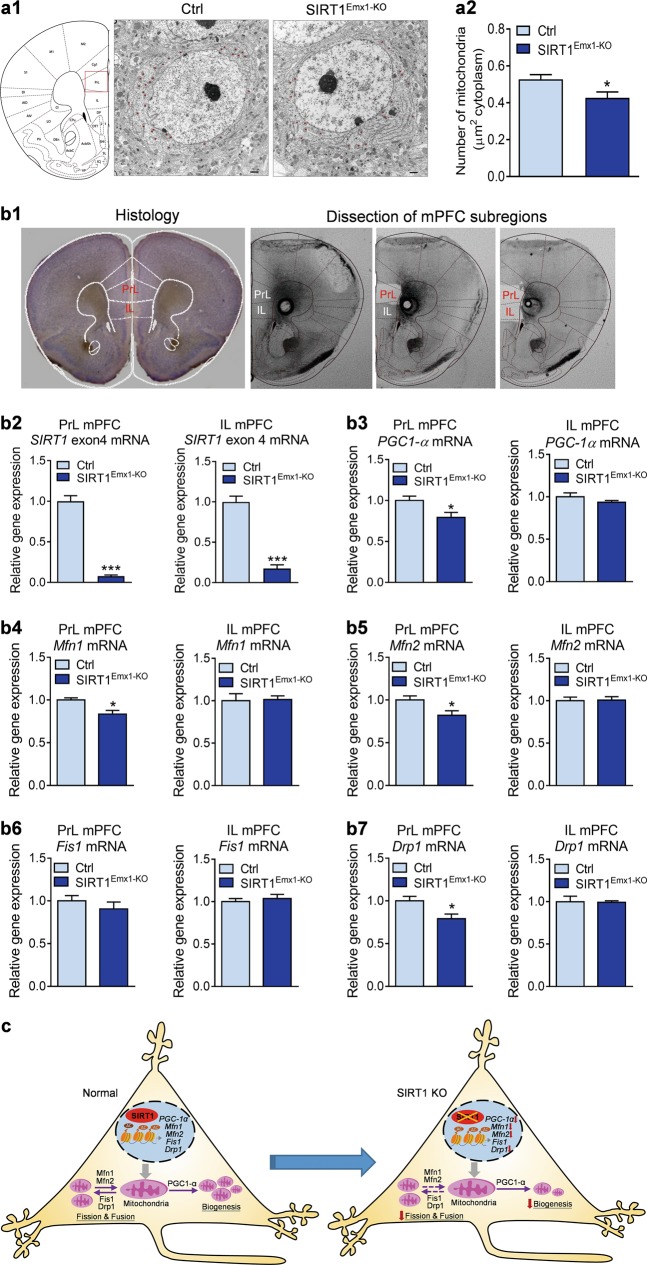
Loss of sirtuin 1 (SIRT1) impairs mitochondrial biogenesis in medial prefrontal cortex (mPFC) neurons. **a1** Representative electron micrographs showing mitochondria in mPFC neurons from male control SIRT1^flox/flox^ (Ctrl) and SIRT1^Emx1-KO^ mice. **a2** Mitochondrial density. The number of mitochondria per unit area (μm^2^) of cytoplasmic soma in each neuron. *n* = 18 neurons from 2 male control SIRT1^flox/flox^ (Ctrl) mice, *n* = 20 neurons from 2 male SIRT1^Emx1-KO^ mice. **b1** Left, histological coronal brain section showing the prelimbic (PrL) and infralimbic (IL) of the mPFC; right, photomicrographs showing dissection of the PrL and IL subregions of mPFC. **b2** SIRT1 exon 4 mRNA. **b3** PGC1-α mRNA. **b4** Mfn1 mRNA. **b5** Mfn2 mRNA. **b6** Fis1 mRNA. **b7** Drp1 mRNA. *n* = 4 male mice per group. **c** Schematic diagram illustrating mitochondrial biogenesis and dynamics in PrL mPFC neurons in response to loss of SIRT1. *P < 0.05, ***P < 0.001 compared with the Ctrl group

We examined the effects of loss of SIRT1 in mPFC neurons on mitochondria machinery. First, mitochondrial density in pyramidal neurons was assessed by electron microscopy. SIRT1^Emx1-KO^ mice showed a decrease in mitochondrial density in mPFC neurons (Fig. 4a2, *t*_(36)_ = 2.443, *P* = 0.020). Next, we determined the expression levels of specific genes involved in mitochondrial biogenesis and dynamics in the subregions of mPFC. As shown in Fig. 4b2, we confirmed the deletion of SIRT1 exon4 in both the prelimbic (*t* test with Welch’s correction, *P* < 0.001) and infralimbic mPFC (*t*_(6)_ = 9.744, *P* < 0.001) of SIRT1^Emx1-KO^ mice. mRNA expression levels of *PGC-1α*, a major regulator of mitochondrial biogenesis, were significantly decreased in the prelimbic (*t*_(6)_ = 2.563, *P* = 0.043), but not infralimbic (*t*_(6)_ = 1.254, *P* = 0.257), mPFC of SIRT1^Emx1-KO^ mice (Fig. 4b3). Furthermore, we examined the expression levels of genes that mediate mitochondrial fusion, including mitofusin 1 (MFN1) and MFN2, and mitochondrial fission, including fission 1 (FIS1) and dynamic-related protein (Drp1) [62]. We found that mRNA levels of *Mfn1* (*t*_(6)_ = 3.297, *P* = 0.017), *Mfn2* (*t*_(6)_ = 2.502, *P* = 0.046) and *Drp1* (*t*_(6)_ = 2.718, *P* = 0.035) were decreased in the prelimbic mPFC but not in the infralimbic mPFC (*Mfn1* (*t*_(6)_ = 0.159, *P* = 0.879; *Mfn2* (*t*_(6)_ = 0.138, *P* = 0.895; *Drp1* (*t*_(6)_ = 0.121, *P* = 0.908). Expression of *Fis1* mRNA was unaltered in either the prelimbic (*t*_(6) _= 0.918, *P* = 0.394) or infralimbic (*t*_(6)_ = 0.610, *P* = 0.564) mPFC (Fig. 4b4–b7). These results suggest that SIRT1 in the prelimbic mPFC is required for normal expression of specific genes that are critical for mitochondrial biogenesis and dynamics.

### Activation of SIRT1 reverses CUS-induced depressive-like behaviors

**Fig. 5:**
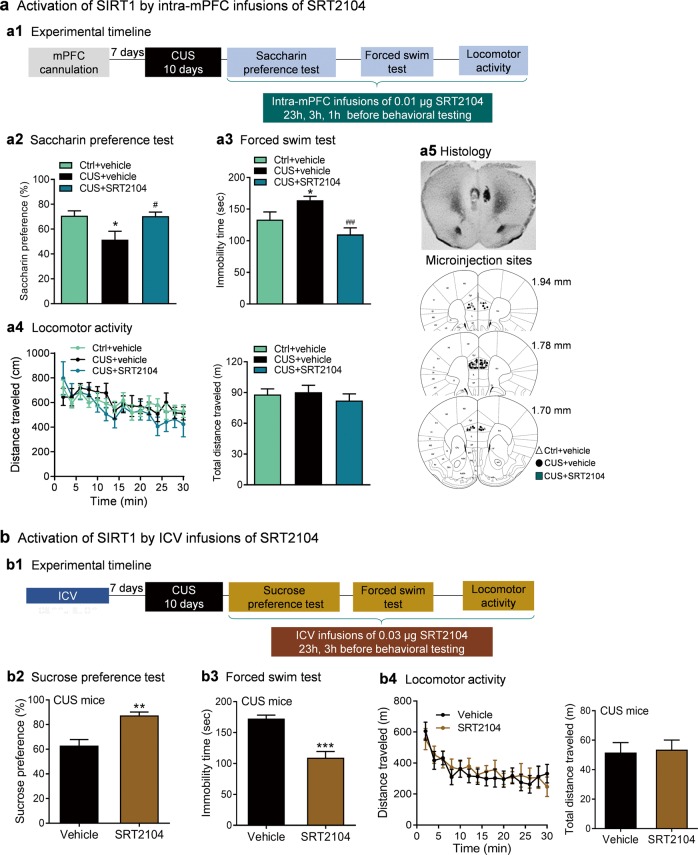
Sirtuin 1 (SIRT1) activation reverses chronic unpredictable stress (CUS)-induced depressive-like behaviors in male wild-type C57BL/6J mice. **a** Activation of SIRT1 by intra-medial prefrontal cortex (intra-mPFC) infusions of SRT2104. **a1** The experimental timeline. **a2** Saccharin preference test. Ctrl+vehicle, *n* = 8; CUS+vehicle, *n* = 10; CUS+SRT2104, *n* = 10. **a3** Forced swim test. Ctrl+vehicle, *n* = 11; CUS+vehicle, *n* = 10; CUS+SRT2104, *n* = 11. **a4** Locomotor activity. Left, time course of locomotor activity over the 30-min test session; right, total distance traveled in 30 min. Ctrl+vehicle, *n* = 11; CUS+vehicle, *n* = 10; CUS+SRT2104, *n* = 9. **a5** Histological verification of intra-mPFC injection sites. Upper, representative image showing the deposition sites within the mPFC; lower, schematic illustration of the intra-mPFC bilateral injection sites. Schematic line drawings of coronal brain sections were adapted from the mouse brain atlas [33]. *P < 0.05 compared with Ctrl+vehicle treatment. ^#^*P* < 0.05, ^###^*P* < 0.001 compared with CUS+vehicle treatment. **b** Activation of SIRT1 by intracerebroventricular infusions of SRT2104. **b1** The experimental timeline. **b2** Sucrose preference test. Vehicle, *n* = 13; SRT2104, *n* = 11. **b3** Forced swim test. **b4** Locomotor activity. Left, time course of locomotor activity over the 30-min test session; right, total distance traveled in 30 min. Vehicle, *n* = 12; SRT2104, *n* = 11. **P < 0.01, ***P < 0.001 compared with vehicle treatment

We next determined whether activation of SIRT1 selectively in the prelimbic mPFC is sufficient to reverse depressive behaviors induced by chronic stress. Mice were cannulated in the prelimbic mPFC and exposed to 10 days of CUS or briefly handled. Then mice received three bilateral infusions of either the SIRT1 activator SRT2104 (0.01 μg) or vehicle into the prelimbic mPFC at 23, 3, and 1 h prior to behavioral testing (Fig. 5a1). The dose was based upon our pilot data. We found that CUS decreased saccharin preference and increased immobility in the forced swim test (Fig. 5a2, a3). These effects of CUS were reversed by intra-prelimbic mPFC injections of SRT2104 (Fig. 5a2, a3; *F*_(2,25)_ = 4.89, *P* = 0.016 for saccharin preference; *F*_(2, 29)_ = 7.002, *P* = 0.003 for forced swim test). SRT2104 had no effect on locomotor activity (Fig. 5a4; distance travelled: time, *F*_(14,378)_ = 6.698, *P* < 0.001; treatment, *F*_(2,27)_ = 0.423, *P* = 0.660; time × treatment, *F*_(28,378)_ = 0.991, *P* = 0.482; total distance: *F*_(2,27)_ = 0.423, *P* = 0.660).

Given the opposing effects of SIRT1 in different brain regions [16, 17], additional studies examined the effects of ICV injection of SRT2104 on CUS-induced depression-related behaviors. ICV cannulated mice were subjected to 10 days of CUS and received two ICV injections of SRT2104 (0.03 μg) or vehicle at 23 and 3 h prior to behavioral testing (Fig. 5b1). Central activation of SIRT1 by ICV infusions of SRT2104 significantly increased sucrose preference (Fig. 5b2, *t*_(22)_ = 3.708, *P* = 0.001) and decreased immobility in the forced swim test (Fig. 5b3, *t*_(21)_ = 5.584, *P* < 0.001) without affecting locomotor activity (Fig. 5b4; distance travelled: time, *F*_(14,294)_ = 10.76, *P* < 0.001; treatment, *F*_(1,21)_ = 0.044, *P* = 0.836; time × treatment, *F*_(14,294)_ = 0.808, *P* = 0.661; total distance: *t*_(21)_ = 0.210, *P* = 0.836).

## Discussion

Our results provide the first evidence that SIRT1, an NAD^+^-dependent class III histone deacetylase, in forebrain excitatory neurons exerts sexually dimorphic effects on depression-like behaviors. We show that ablation of SIRT1 in cortical and hippocampal glutamatergic neurons results in depression-like behaviors in male but not in female mice. A similar phenotype was induced by selective ablation of SIRT1 in the mPFC of adult male mice. Furthermore, loss of SIRT1 in layer V pyramidal neurons of the prelimbic, but not infralimbic, mPFC reduces neuronal excitability and excitatory synaptic transmission. Activation of SIRT1 in the prelimbic mPFC is sufficient to reverse chronic stress-induced depressive behaviors. These results suggest that SIRT1 activity in mPFC pyramidal neurons is a key player in the modulation of depression-related behaviors.

Major depression is a clinically and genetically heterogeneous disorder. It has been difficult to identify genetic risk variants for major depression. SIRT1 is one of the first two genes linked to major depression, which has been revealed in both genders and different populations [6–8]. However, inconsistent results in animal models have been reported on the behavioral effects of SIRT1. Libert and colleagues first reported that brain-specific SIRT1 knockout mice were resilient to depression, whereas mice with global SIRT1 overexpression were more susceptible to depression, based upon the forced swim and sucrose preference test results [18]. However, in this study brain-specific SIRT1 knockout and whole-body SIRT1 overexpression caused severe hyperlocomotion and hypolocomotion in mice, respectively. These effects on locomotor activity can greatly confound the interpretations of behavioral data from the forced swim test [18]. Additionally, those brain-specific SIRT1 knockout mice showed a drastic reduction in sucrose preference under non-stressed condition and exhibited no further decrease after social defeat stress [18], which could reflect a floor effect reducing sucrose preference rather a resilience response to stress. Other investigations by targeting SIRT1 in particular brain regions suggest that the effects of SIRT1 activity on depression-like behaviors may be brain-region dependent [16, 17, 20]. Abe-Higuchi et al. found that activation of SIRT1 in the dentate gyrus (DG) by local infusion of SRT2104 using osmotic minipumps for 2 weeks blocked the repeated restraint stress-induced decreases in social interaction and sucrose preference, whereas inhibition of SIRT1 increased depressive behaviors [17]. In contrast, Ferland et al. demonstrated that intra-DG infusion of a SIRT1 inhibitor prevented chronic stress-induced anhedonia in rats [20]. On other hand, Kim et al. found that pharmacological inhibition of SIRT1 or SIRT1 knockdown in the nucleus accumbens produced antidepressant-like effects and blocked social defeat-induced social avoidance, whereas overexpression of SIRT1 was pro-depressive [16]. These discrepancies may be due to the differences in animal species, genetic background, behavioral procedures, and methods used to manipulate SIRT1 activity or expression. Of note, in all these studies mentioned above, only male animals were used for the experiments. In the present study, we addressed the specific role of SIRT1 in forebrain glutamatergic neurons in depressive behaviors in both male and female mice. We found that loss of SIRT1 in these neurons led to depressive behaviors, including anhedonia and behavioral despair, in male but not in female mice. In contrast to the whole-brain SIRT1 knockout [18], both male and female mice with SIRT1 deletion in forebrain glutamatergic neurons showed normal locomotor activity. The absence of a depressive phenotype in female mice was unexpected given that the genetic association between SIRT1 and major depression was first identified in a large population of female patients [6]. This could be due to sexual dimorphism in the density of neurons, dendritic complexity, and synaptic density in cortical areas [63–66]. It has been reported that female brains have lower numbers of neurons and synaptic density in the neocortex including the PFC [63–65]. Recent brain-wide mapping revealed sexual dimorphism in subcortical inhibitory neurons [67]. In contrast to cortical areas, female mouse brains have more inhibitory neurons in most subcortical regions [67]. We speculate that different neuronal populations in cortical and subcortical regions may be involved in mediating sex differences in SIRT1 action on depressive behaviors.

The PFC has an important role in the pathogenesis of major depressive disorder. Dorsolateral PFC hypoactivation has long been a recognized concomitant of depression. The severity of depression correlates with the degree of PFC inactivity; such inactivity can be normalized by antidepressant treatment [68–72]. The rodent mPFC is thought to be analogous to the primate dorsolateral PFC [73, 74]. It has been reported that electrical or optogenetic stimulation of the mPFC exert antidepressant-like effects, reducing behavioral despair in the forced swim test [75] and alleviating chronic unpredictable/mild stress-induced anhedonia and chronic social defeat-induced social avoidance [76–78]. In this study, we found that AAV-mediated delivery of Cre resulted in SIRT1 knockdown in the mPFC and induced depressive behaviors, indicating the importance of SIRT1 action on mPFC neurons in mood regulation. It is noteworthy that the AAV vector used in this study has the nonspecific CMV promoter driving expression of Cre recombinase expression; thus SIRT1 knockdown may occur in both mPFC glutamatergic and non-glutamatergic neurons. Pyramidal output neurons in the mPFC, which are excitatory glutamatergic neurons and reside in the layer V, integrate and transfer information from extra-cortical inputs and local circuits to other cortical areas and subcortical structures [79–81]. The evidence supporting the involvement of layer V pyramidal neurons in mood-related behaviors comes from the studies showing that direct optogenetic stimulation of layer V pyramidal neurons produces robust antidepressant-like effects [58]. To investigate whether hypoactivity of layer V pyramidal neurons may underlie the depressive phenotype observed in mice lacking SIRT1 in mPFC neurons, whole-cell patch-clamp recordings were made from neurons in both the prelimbic and infralimbic mPFC. Interestingly, loss of SIRT1 decreased intrinsic neuronal excitability in pyramidal neurons in the prelimbic, but not iinfralimbic, mPFC. Given that layer V pyramidal neurons receive long-range and local excitatory synaptic inputs [82, 83], we further examined whether loss of SIRT1 would affect excitatory synaptic transmission. Both the frequency and amplitude of sEPSCs were decreased in layer V pyramidal neurons lacking SIRT1 in the prelimbic mPFC, reflecting decreased probability of glutamate release from presynaptic terminals as well as reduced postsynaptic responsiveness to glutamate. sEPSCs comprise AP-dependent and -independent synaptic events. After blocking APs with TTX, the decrease in excitatory synaptic transmission was abolished, suggesting that the changes in synaptic events caused by loss of SIRT1 are AP-dependent. In contrast, pyramidal neurons in the infralimbic mPFC did not show any significant change in either the frequency or amplitude of sEPSCs. Since SIRT1 is expressed in the prelimbic and infralimbic subdivisions with comparable levels, the differences in excitability and synaptic activity in pyramidal neurons between these two subdivisions are unlikely to be due to expression of SIRT1. Based upon our results, we postulate that loss or reduction of SIRT1 activity may contribute to hypofunction of the PFC, leading to the development of depression in males. Conversely, we showed that activation of SIRT1 in the prelimbic mPFC reversed CUS-induced anhedonia and behavioral despair. These data implicate the involvement of mPFC SIRT1 in both the pathogenesis and treatment of depression.

Neurons depend on mitochondrial function to maintain normal neuronal excitability and execute the complex processes of synaptic transmission and plasticity [84]. Consistent with hypoexcitability of pyramidal neurons observed in the prelimbic mPFC of SIRT1^Emx1-KO^ mice, we found that loss of SIRT1 reduced the expression levels of genes involved in mitochondrial biogenesis and dynamics in the prelimbic but not infralimbic mPFC. An important major target for SIRT1 deacetylation activity is PGC-1α, which acts as a master regulator of mitochondrial biogenesis and function [85]. Acetylation of PGC-1α inhibits its transcriptional activity [86]. SIRT1 directly affects the transcriptional activity of PGC-1α through deacetylation [87] and subsequent expression of its target genes involved in mitochondrial biogenesis and function [88–91]. Both SIRT1 and PGC-1α were not only found in the nucleus but also in the mitochondria associated with the mitochondrial DNA nucleoids [92]. We demonstrated that ablation of SIRT1 gene decreased mitochondrial density in mPFC neurons and reduces PGC-1α gene expression in the prelimbic mPFC. This suggests that the reduction of mitochondrial biogenesis caused by loss of SIRT1 may involve both the increased acetylation status and the reduced expression levels of PGC-1α. These findings provide evidence to support the idea that mitochondrial dysfunction in the prelimbic mPFC may contribute to the development of depression symptoms.

These results indicate that SIRT1 activity in mPFC neurons is a key regulator of sex-specific depression-related behaviors. In line with the functional difference between prelimbic and infralimbic mPFC [61, 78, 93–95], our data suggest abnormal SIRT1 activity and impaired neuronal excitability in prelimbic pyramidal neurons may contribute to the development of depression. Further studies are needed to identify neuronal populations and neural circuits mediating sex difference in SIRT1 action on depression-related behaviors.
